# Tampa Scale of Kinesiophobia may underestimate task-specific fear of movement in people with and without low back pain

**DOI:** 10.1097/PR9.0000000000001081

**Published:** 2023-06-07

**Authors:** Liam-Pierre Mathieu Tissot, David William Evans, Edward Kirby, Bernard Xian Wei Liew

**Affiliations:** a School of Sport, Rehabilitation and Exercise Sciences, University of Essex, Colchester, Essex, United Kingdom; b Centre of Precision Rehabilitation for Spinal Pain, School of Sport, Exercise and Rehabilitation Sciences, University of Birmingham, Edgbaston, Birmingham, United Kingdom; c Musculoskeletal Physiotherapy, Essex Partnership University NHS Foundation Trust, Runwell, Wickford, United Kingdom

**Keywords:** Low back pain, Psychology, Psychometrics, Fear, Fear of movement, Lifting

## Abstract

**Introduction:**

The Tampa Scale of Kinesiophobia (TSK) is commonly used to assess fear of movement (FoM) in people with low back pain (LBP). However, the TSK does not provide a task-specific measure of FoM, whereas image-based or video-based methods may do so.

**Objectives:**

To compare the magnitude of FoM when assessed using 3 methods (TSK-11, image of lifting, video of lifting) in 3 groups of people: current LBP (LBP), recovered LBP (rLBP), and asymptomatic controls (control).

**Methods:**

Fifty-one participants completed the TSK-11 and rated their FoM when viewing images and videos depicting people lifting objects. Low back pain and rLBP participants also completed the Oswestry Disability Index (ODI). Linear mixed models were used to estimate the effects of methods (TSK-11, image, video) and group (control, LBP, rLBP). Linear regression models were used to assess associations between the methods on ODI after adjusting for group. Finally, a linear mixed model was used to understand the effects of method (image, video) and load (light, heavy) on fear.

**Results:**

In all groups, viewing images (*P* = 0.009) and videos (*P* = 0.038) elicited greater FoM than that captured by the TSK-11. Only the TSK-11 was significantly associated with the ODI (*P* < 0.001). Finally, there was a significant main effect of load on fear (*P* < 0.001).

**Conclusion:**

Fear of specific movements (eg, lifting) may be better measured using task-specific measures, such as images and videos, than by task-generic questionnaires, such as the TSK-11. Being more strongly associated with the ODI, the TSK-11 still plays an important role in understanding the impact of FoM on disability.

## Abstract 2

Supplemental Digital Content is Available in the Text.

## 1. Introduction

Individuals who experience low back pain (LBP) often associate bending or lifting activities with pain and avoid such movements, developing a conditioned fear of movement (FoM).^10,14,49^Commonly termed as “kinesiophobia,” FoM reflects “an excessive, irrational, and debilitating fear of physical movement and activity that results from a feeling of vulnerability in regard to a painful injury or reinjury.”^25^ Avoidant behaviors resulting from FoM are important contributors to the delayed recovery of LBP patients and the development of chronic LBP.^50^ Typically, FoM is measured using patient-reported outcome measures (PROMs), such as the Tampa Scale for Kinesiophobia (TSK).^51^ However, the TSK can be criticized for its use of items unrelated to movement (eg, “People aren't taking my medical condition seriously enough”).^47^ Such items raise the question as to its face validity when assessing FoM. Several studies have even reported weak correlations between physiological measures of fear responses (eg, changes in skin conductance and blink startle response) and PROMs.^5,19,40,44^ Consequently, Caneiro et al.^5^ suggested the possibility that a description of a feared movement does not elicit the same physiological fear responses as when tasked with actually performing the movement, which may be a limitation of the TSK and other fear-related PROMs.

Compared with PROMs with text-only items, image-based items can visually depict specific movements known to load the lower back and may be better suited for eliciting a FoM response. For example, the Photograph Series of Daily Activities (PHODA) is an image-based questionnaire originally designed to assess FoM with daily activities for LBP patients.^27,36,45^ The PHODA has been believed to provide a more valid measure of FoM than PROMs,^45^ and thus, it provides a more clinically specific method of assessing outcomes associated with treatments targeting FoM. The face validity of images comes into question when we consider that an image of a movement is static and thus conveys a stationary posture. Hence, PROMs that use static images like the PHODA may be measuring fear of a posture rather than a movement. Investigating this, Perez-Fernandez et al.^38^ noted no significant difference in the use of images when compared with videos of common therapeutic exercises for low back pain. However, there is indirect evidence suggesting that viewing images and sounds in video sequences induce stronger and more accurate fear and happiness responses than images alone in healthy adults.^22^ In addition, Courtney et al.^11^ found that computer-generated videos better elicited physiological fear responses when compared with still images and emphasized the importance of motion in the elicitation of fear responses.

To our knowledge, no studies have directly compared traditional PROMs with image-based and video-based methods of measuring FoM in people across the LBP recovery spectrum. If fear is rated differently in video-based methods than the other 2 methods, the superior face validity of video-based methods would be a reason to suggest that they be used to assess FoM. Therefore, this study aimed to compare written, image-based, and video-based questionnaires as tools for measuring LBP-related FoM. Because fear can persist beyond symptom recovery in people with LBP,^42^ we also wanted to investigate the effect of currently experiencing pain vs having recently experienced pain (in the previous 6 months but not in the past month), vs being asymptomatic for the past 6 months.

## 2. Methods

Based on the above aims, our first hypothesis was that individuals currently experiencing LBP will report the greatest fear in the video method, whilst asymptomatic individuals will report the lowest fear using the written method (an interaction between group and method). For our second hypothesis, we expected fear to be most predictive of disability when using the video method, and least predictive of disability when using the written method. Our third hypothesis was that viewing a person lifting the heaviest load will evoke the greatest fear and viewing a person lifting the lightest load will evoke the least fear.

### 2.1. Study design and participants

This was a cross-sectional study in which data were collected through an online survey built using Qualtrics XM (Qualtrics, Provo, UT). Recruitment occurred between November 2021 and July 2022. Individuals aged 18 years and older were recruited through convenience sampling (flyers, posters, and social media promotion through Instagram, Twitter, and SurveyCircle). Three groups of participants were recruited. In the asymptomatic group (control), participants must not have experienced LBP within the past 6 months. In the current LBP group (LBP), participants must have reported current pain in the lower back lasting longer than 24 hours with an intensity greater than 2/10, as measured on a 0 to 10 numerical rating scale (0 = no pain, 10 = maximal pain). Participants in the recovered group (rLBP) must have been asymptomatic for a continuous period of at least 1 month^21^ following an episode of LBP experienced within 6 months of recruitment. Participants were excluded if LBP was due to medical conditions, including spinal fracture, rheumatological conditions, metabolic conditions, infectious conditions, cancer, kidney conditions, or gastrointestinal conditions. Written informed consent was gained from all participants before study enrollment. Ethical approval was received from the University of Essex Human Research Ethics Committee (ETH2122-0276).

### 2.2. Sample size

A previous study reported a correlation magnitude between picture-based and questionnaire-based methods of measuring fear of 0.6.^5^ To detect a correlation (Pearson *r*) of 0.6 at a statistical power of 0.8 and alpha of 0.05 for each group, 19 participants needed to be recruited per group, resulting in a predicted sample size of 57. Within the available recruitment window, this study only recruited 51 participants who completed the survey.

### 2.3. Questionnaires

In addition to the collection of basic demographic characteristics (age, gender, height, and weight), the survey consisted of 3 sections. In the first section, all participants were presented with the TSK-11 to assess their current FoM.^51^ The TSK-11 has demonstrated good internal consistency with Cronbach α ranging from 0.79 to 0.81.^20,43,51^ Several studies^43,51^ have reported a moderate yet significant inverse association between the TSK-11 and pain acceptance measures, indicating good construct validity. A previous study reported a meaningful clinical change score of 4 points,^51^ which is equivalent to a 21% change using the 0 to 100 scaling method reported below in the statistical analysis section. Overall, the TSK-11 is considered a valid and reliable tool for assessing FoM.^20,43,51^ A previous study defined categories as “minimal” (TSK-11 ≤ 22), “low” (TSK-11 = 23–28), “moderate” (TSK-11 = 29–35), and “high” fear (TSK-11 ≥ 36).^7^

In the second section, all participants completed 7 single-item questions that required participants to rate their fear levels towards images or videos depicting various lifting activities (see supplementary materials, available at http://links.lww.com/PR9/A196): 4 images (2 heavy, 2 light) and 3 videos (2 light, 1 heavy) depicting either a male (21 years, 178 cm, 82 kg) or female (21 years, 160 cm, 45 kg) lifting an empty 40 L laundry basket or two 24 kg adjustable dumbbells (Bowflex, Nautilus Inc, Vancouver, WA). Lifts were performed at a fixed rate of 45 beats per minute (“beat” fully bent, “beat” upright) using an auditory metronome. To avoid unintended influence of observed body language and facial expressions,^37,46^ these were kept neutral. Photographs and videos of these activities were captured using a tripod-mounted iPad (Apple, Cupertino, CA). The sequence of presentation of images and videos was randomized using Qualtrics XL software. The format of the associated questions was based on that of the PHODA, where subjects rated their perceived harmfulness of the displayed activities on a scale of 0 to 100 (0 = not harmful, 100 = extremely harmful).^27,35,36^ We chose to depict lifting because it is the most studied activity regarding LBP^34^ and is often included in pictorial questionnaires assessing fear of pain and movement.^27,47^ In addition, bending or lifting with a rounded back is often considered a dangerous movement and plays an important role in FoM for individuals with LBP.^5^

The last section required only LBP and rLBP participants to complete the Oswestry Disability Index (ODI) to assess the interference of their LBP with activities of their daily life.^15^ The ODI has been shown to have good internal consistency with Cronbach α ranging from 0.71 to 0.87^15,48^ and excellent construct validity being particularly good at discerning levels of disability for severely affected patients.^39,48^ Overall, the ODI is considered an acceptable tool for assessing the level of disability caused by LBP in the general population.^15,39,48^

### 2.4. Statistical analysis

All scales were converted to a common 0 to 100 scale for comparison and analysis, which is a common practice when combining data sets from different study cohorts for analysis.^12^ Tampa Scale of Kinesiophobia-11 items are each rated on a 1 to 4 scale, producing a minimum summed score of 11 (indicating no FoM) and a maximum score of 44 (indicating highest FoM). The TSK-11 was converted to a 0 to 100 scale using the following formula: ([reported score − 11]/[maximum possible score − 11]) × 100. Oswestry Disability Index items are each rated on a 0 to 5 scale, with a minimum summed score of 0 indicating no disability and a maximum score of 50 indicating complete disability. The ODI was converted to a 0 to 100 scale using the following formula: (reported score/maximum possible score) × 100. For each participant, the values of the 2 heavy images, 2 light images, and 2 light videos were averaged to produce a single fear score for a heavy image, light image, and light video, respectively. Statistical analyses were performed using SPSS Statistics, version 28 (IBM, Armonk, NY).

For the first hypothesis, that individuals experiencing LBP will report the greatest fear in the video method, whilst asymptomatic individuals will report the lowest fear using the written method; a linear mixed model was used to estimate the effects of methods (TSK-11, image, video), group (control, LBP, rLBP), and the interaction between methods and group on the outcome value of the fear outcome that was scaled to 0 to 100. Estimated marginal means were used for post hoc pairwise contrast in the event of statistical significance in the main and interaction effects. For the second hypothesis, that fear will be most predictive of disability using the video method, and least predictive of disability when using the written method; 3 linear regressions were used to assess the predictive power of methods (TSK-11, image, video) on disability (ODI score) after adjusting out the effect of group (control, LBP, rLBP). For the third hypothesis, that viewing a person lifting the heaviest load will evoke the greatest fear and viewing a person lifting the lightest load will evoke the least fear, a linear mixed model was used to understand the effects of the method (image, video) and load (light, heavy) on fear, after adjusting out the effect of group (control, LBP, rLBP). The statistical assumptions of normality and homogeneity of variance were assessed using residual diagnostic plots. Statistical significance was determined using a threshold of *P* < 0.05.

## 3. Results

**Figure 1.**
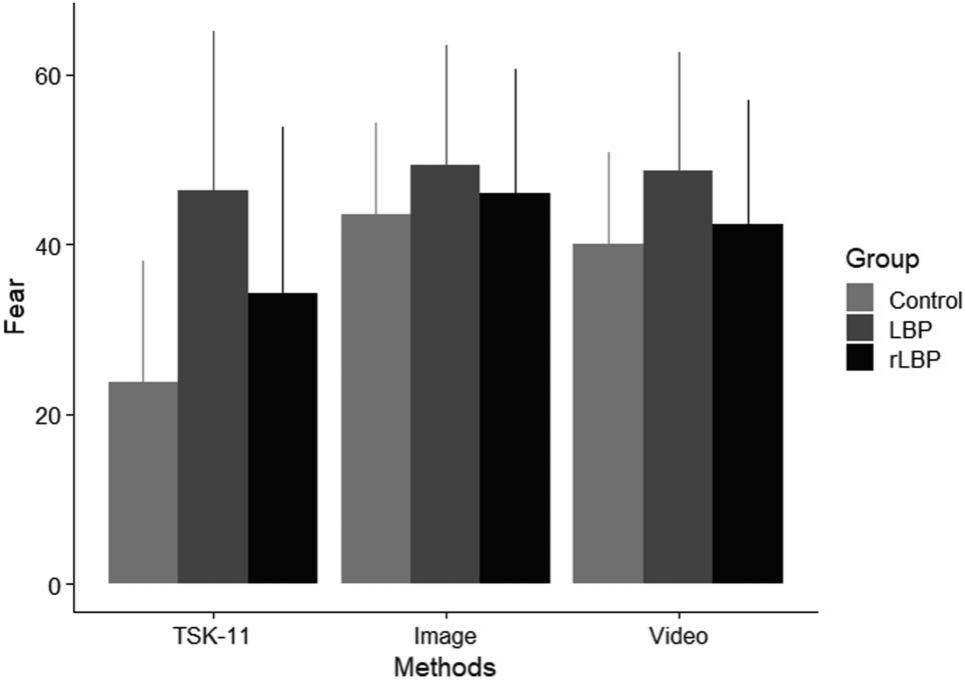
Mean (with 95% confidence interval error bars) fear levels reported using different methods in people with LBP, those who recently recovered from LBP, and asymptomatic controls. LBP, low back pain; rLBP, recovered low back pain; TSK, Tampa Scale of Kinesiophobia.

Descriptive statistics of the included participants are displayed in Table 1. Control and rLBP participants had minimal to low levels of fear,^7^ whilst LBP participants had low to moderate levels of fear.^7^ For all 3 hypotheses, a detailed report of our regression analysis parameter estimates can be found in the supplementary material (available at http://links.lww.com/PR9/A196). For the first hypothesis that individuals experiencing LBP will report the greatest fear and asymptomatic individuals will report the lowest fear, there was no significant interaction between group and method, F(4,95) = 0.848, *P* = 0.498 (Fig. 1). The main effect of group on fear was not significant, F(2,44) = 1.03, *P* = 0.367. The main effect of method on fear was significant (F(2,91) = 3.65, *P* = 0.030). Post hoc analysis showed that images (mean difference = 11.51 (95% CI [2.96, 20.05]), *P* = 0.009) and videos (mean difference = 8.95 (95% CI [0.50, 17.39]), *P* = 0.038) elicited greater fear than the TSK-11.

**Table 1:** Descriptive statistics of participants.

Group	Control (n = 24)	LBP (n = 14)	rLBP (n = 13)
Gender			
Male	14 (26.92)	10 (19.23)	7 (13.46)
Female	10 (19.23)	3 (5.77)	5 (9.62)
Other	—	1 (1.92)	1 (1.92)
Age (y)	29.71 (11.75)	28.29 (11.32)	35.46 (13.47)
Height (cm)	175.96 (11.22)	183.15 (14.22)	175.15 (9.87)
Weight (kg)	74.97 (13.37)	86.61 (16.21)	75.69 (14.34)
Pain intensity (range 0–10)	—	4.07 (2.37)	—
TSK-11 (range 11–44)	18.83 (4.00)	26.29 (7.19)	22.31 (5.50)
ODI (range 0–50)	—	13.71 (11.28)	4.15 (3.21)

Gender presented as n (%). All other values are provided as M (SD).

LBP, low-back pain; M, mean; ODI, oswestry disability index; rLBP, recovered from low-back pain; TSK-11, Tampa Scale for Kinesiophobia, 11-item version.

For the second hypothesis that fear will be most predictive of disability using the video method and least predictive of disability when using the written method, the overall regression for images was significant (*R*^2^ = 0.26, F(2, 24) = 4.25, *P* = 0.026) but self-reported fear on image-based questions was not significantly associated with disability (*B* = 0.05, *P* = 0.725). Second, the overall regression for videos was significant (*R*^2^ = 0.26, F(2, 24) = 4.17, *P* = 0.028), but self-reported fear on video-based questions was not significantly associated with disability (*B* = 0.01, *P* = 0.933). Third, the overall regression for the TSK-11 was significant (*R*^2^ = 0.61, F(2, 24) = 19.07, *P* < 0.001), and self-reported fear of the TSK-11 was significantly associated with disability (*B* = 0.60, *P* < 0.001).

**Figure 2.**
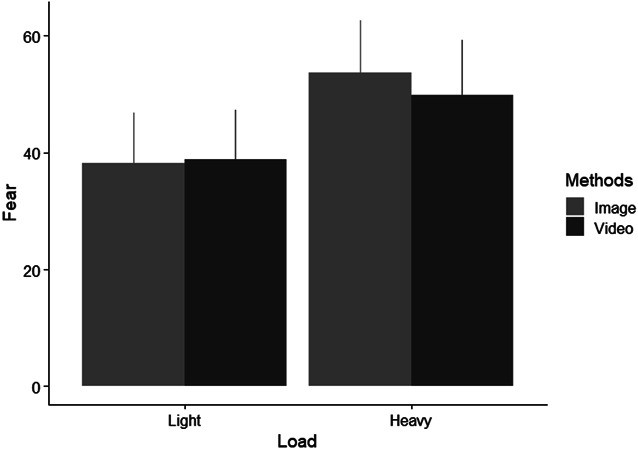
Mean (with 95% confidence interval error bars) fear levels reported when viewing 2 different loads in image and video format.

For the third hypothesis that viewing a person lifting the heaviest load will evoke the greatest fear and viewing a person lifting the lightest load will evoke the least fear, there was no significant interaction between method and load, F(1,137) = 1.11, *P* = 0.294 (Fig. 2). The main effect of method on fear was not significant (F(1,137) = 0.52, *P* = 0.472). However, the main effect of load on fear was significant (F(1,137) = 37.56, *P* < 0.001). Post hoc analysis shows that depictions of higher loads (mean difference = 13.23 (95% CI [8.97, 17.50]), *P* < 0.001) elicited greater fear than depictions of lighter loads.

## 4. Discussion

This study aimed to assess the impact of different self-reported methods (written descriptions, static images, or videos) on the assessment of FoM in individuals across a spectrum of LBP states. Contrary to our first hypothesis that there would be an interaction between group and methods in their effects on fear, the written method resulted in the lowest fear score across all groups, compared with the image and video methods. In contrast to our second hypothesis that fear will be most predictive of disability using the video method and least predictive of disability when using the written method, the TSK-11 was most predictive of disability, whereas the images and videos depicting lifting were not predictive of disability. Finally, our findings supported our third hypothesis that viewing individuals lifting heavier loads would evoke greater fear than lighter loads.

Our result of a significant difference between TSK-11 and image-based scores is partially supported by the current literature; a previous study reported a moderate positive correlation between PHODA and TSK-17 scores,^27^ whereas other studies found no significant correlation.^13,33,35^ However, the difference in scaled fear scores between the TSK-11 and image/video methods in this study ranged from 9% to 11.5%, which is less than the scaled clinical meaningful change TSK-11 threshold of 21%.^51^ Variation in findings could be due to this study correlating perceived fear on a single activity to the latent fear measured by the TSK-11, whilst other studies correlated the mean fear score across all activities within PHODA to different TSK variants.^13,27,33,35^ There was a nonstatistically significant trend that the more severe the LBP status, the more the TSK underestimates fear relative to image/video methods. For example, TSK-11 underestimated fear in controls by 19.8%, in rLBP by 11.7%, and in LBP by 3.0% scaled points, relative to an image (Fig. 1). The present findings suggest that the TSK-11, and likely other TSK variants, should not be used in isolation to determine fear of lifting in pain-free cohorts,^23^ within the context of primary prevention medicine^17^ and clinical education.^29^

Although the average difference may not be clinically significant, it is useful to explore the mechanisms underpinning the different methods of assessing fear. Images and videos may be measuring distinct constructs of pain-related fear compared with the TSK.^33^ Meier et al.^32^ reported a correlation between FoM, measured using a similar video-based approach, and brain activity in fear-related cortical regions, such as the amygdala and the insula. However, TSK-11 scores were more related to the cortical activity of the orbital frontal context, indicating that TSK-11 score may better reflect anxiety.^33^ In addition, FoM has been shown to affect motor strategies in people with LBP.^9,16^ Given that simply observing movements can activate cortical motor regions involved during movement^1^ and that cortical motor changes may be associated with the presence of LBP,^6^ observing images or videos may activate a greater portion of the cortical region involved in FoM compared with a written questionnaire such as the TSK-11. Further research into how differences in cortical activation patterns associated with fear when observing movement and different static postures could shed light on the neural substrates associated with FoM.

We found that the TSK-11 was predictive of disability, whereas image-based and video-based scores were not. The TSK-11 and the ODI are task-generic questionnaires that do not measure fear and disability on a single lifting task.^15,51^ By contrast, the image and video items are task specific, measuring FoM concerning just a single lifting task. It may be that task-generic questionnaires are more likely to be associated with each other and less associated with task-specific questionnaires. One study^30^ found that fear reported on task-specific PHODA images (eg, lifting a pot with a bent back or shoveling dirt) significantly predicted objective lumbar range of motion (ROM), whereas nonspecific PHODA images were not predictive of lumbar ROM. Another study^28^ reported that lifting-specific PHODA items were associated with altered lumbar extensor muscle activity, whereas total PHODA scores were not. Interestingly, another study^24^ reported that both the lifting-specific PHODA items were not associated with objective lumbar ROM, but this was conducted in pain-free adults.

There was no significant difference in self-reported fear when comparing image-based questions with video-based questions. The present study, like that of Perez-Fernandez et al.,^38^ included images and videos depicting activities(s) with a controlled tempo and neutral facial expressions. However, Pérez-Fernandez et al.^38^ did not assess fear during lifting. Despite the different approaches to depicting lifting, both this study and that of Pérez-Férnandez et al.^38^ reported no significant difference between images and videos when assessing FoM in LBP patients. Participants watching the videos may focus solely on the posture depicted by our image which, given the high spinal flexion angle and external lever arm of the load from the spine, is known to impose the greatest spinal load.^8^ Put simply, participants appear to automatically focus their attentional resources on the posture(s) perceived to be most dangerous to the back, whilst ignoring other postures in the sequence of movement. Further research investigating differences between FoM assessed using a video, and different postures across the spectrum of motion would be useful.

Self-reported fear was significantly higher when viewing an individual lifting a heavier load than a lighter load, suggesting that our participants perceived heavier loads to be more associated with harm, regardless of LBP status or method (ie, viewing images or videos). It is a common belief that lifting with lumbar flexion is dangerous^4,26^ and that increasing the load when lifting with a flexed lumbar would only increase the risk of injury.^8,31^ Hence, individuals lifting an unknown load tend to assume that the weight is heavy and lift accordingly.^3^ If the load is lighter than expected, it results in a jerk-like motion with an unnecessarily large low back flexion-extension moment,^3^ which may indicate a conditioned fear of damaging the lower back when lifting heavy loads without preparation. Moreover, lifting greater weight causes increased mechanical tension and fatigue, which is associated with decreased knee and hip flexion and greater lumbar flexion during repetitive lifting tasks.^41^

The selection of outcome measures to be used in a busy clinical environment depends not only on their psychometric properties but also on factors such as time and their ability to guide treatment decision making. In patients with a current episode of LBP, this study suggests that a quicker image-based/video-based method could provide the same index of fear as assessed by the more time-consuming TSK-11. Our results also suggest that clinicians may choose to select images/videos depicting a physically more strenuous task to elicit a greater perception of fear than images/videos depicting a less strenuous task.

Our study included several limitations. First, unlike multi-item measures like the TSK-11, the reliability of single-item measures cannot be measured in cross-section studies.^2^ Second, we powered this study based on a prior correlation between 2 methods of fear measurements. There is a potential that this study was underpowered to detect small interaction effect sizes between groups and methods. Third, this study did not assess other types of movement that may elicit fear, such as carrying an object over a certain distance, lifting an object overhead, pushing, or pulling. The PHODA could guide the selection of movements to be depicted in video format, to develop a video database for ease of use in future studies. Fourth, membership in our asymptomatic control group was based entirely on not having suffered from LBP within the past 6 months. We did not investigate participants' LBP histories beyond this period. It is plausible that significant or repeated LBP experienced prior to these 6 months could have induced a lasting FoM. Lastly, our LBP participants had lower levels of fear than some LBP cohorts investigated.^18^ However, the mean unscaled TSK-11 score of 26.29 (SD: 7.19) in our LBP group was very similar to that measured in a study of chronic pain patients (mean: 27.3, SD: 6.1),^43^ suggesting that our convenience sampling approach was adequate. The low to moderate TSK-11 score in this study could exert a “floor” effect that precluded finding a group-by-method interaction. Future studies should explore the differences in fear reporting using different methods in patients with higher levels of fear.

## 5. Conclusions

Fear of specific movements (eg, lifting) may be better measured using task-specific measures, such as images and videos depicting the task, than by task-generic FoM measures such as the TSK-11. This may be more important in patients with milder pain and asymptomatic controls. The TSK-11 is more strongly associated with pain-related disability and therefore still plays an important role in understanding the impact of FoM on disability.

## Disclosures

The authors have no conflict of interest to declare.

## Appendix A. Supplemental digital content

Supplemental digital content associated with this article can be found online at http://links.lww.com/PR9/A196.

## Supplementary Material

SUPPLEMENTARY MATERIAL

## References

[1] Alaerts K Swinnen SP Wenderoth N. Is the human primary motor cortex activated by muscular or direction-dependent features of observed movements? Cortex 2009;45:1148–55. 19100971 10.1016/j.cortex.2008.10.005

[2] Allen MS Iliescu D Greiff S. Single item measures in psychological science. Eur J Psychol Assess 2022;38:1–5.

[3] Butler D Andersson G Trafimow J Schipplein O Andriacchi T. The influence of load knowledge on lifting technique. Ergonomics 1993;36:1489–93. 8287855 10.1080/00140139308968016

[4] Caneiro J O'Sullivan P Smith A Ovrebekk IR Tozer L Williams M Teng MLW Lipp OV. Physiotherapists implicitly evaluate bending and lifting with a round back as dangerous. Musculoskelet Sci Pract 2019;39:107–14. 30553986 10.1016/j.msksp.2018.12.002

[5] Caneiro JP O'Sullivan P Smith A Moseley GL Lipp OV. Implicit evaluations and physiological threat responses in people with persistent low back pain and fear of bending. Scand J Pain 2017;17:355–66. 29031589 10.1016/j.sjpain.2017.09.012

[6] Chang W-J O'Connell NE Beckenkamp PR Alhassani G Liston MB Schabrun SM. Altered primary motor cortex structure, organization, and function in chronic pain: a systematic review and meta-analysis. J Pain 2018;19:341–59. 29155209 10.1016/j.jpain.2017.10.007

[7] Chimenti RL Post AA Silbernagel KG Hadlandsmyth K Sluka KA Moseley GL Rio E. Kinesiophobia severity categories and clinically meaningful symptom change in persons with achilles tendinopathy in a cross-sectional study: implications for assessment and willingness to exercise. Front Pain Res (Lausanne) 2021;2:739051. 35295417 10.3389/fpain.2021.739051 PMC8915659

[8] Cholewicki J McGill SM Norman RW. Lumbar spine loads during the lifting of extremely heavy weights. Med Sci Sports Exerc 1991;23:1179–86. 1758295

[9] Christe G Crombez G Edd S Opsommer E Jolles BM Favre J. Relationship between psychological factors and spinal motor behaviour in low back pain: a systematic review and meta-analysis. PAIN 2021;162:672–86. 33591109 10.1097/j.pain.0000000000002065

[10] Constantinou E Purves KL McGregor T Lester KJ Barry TJ Treanor M Craske MG Eley TC. Measuring fear: association among different measures of fear learning. J Behav Ther Exp Psychiatry 2021;70:101618. 33039814 10.1016/j.jbtep.2020.101618 PMC7689577

[11] Courtney CG Dawson ME Schell AM Iyer A Parsons TD. Better than the real thing: eliciting fear with moving and static computer-generated stimuli. Int J Psychophysiol 2010;78:107–14. 20600370 10.1016/j.ijpsycho.2010.06.028

[12] da Silva T Macaskill P Kongsted A Mills K Maher CG Hancock MJ. Predicting pain recovery in patients with acute low back pain: updating and validation of a clinical prediction model. Eur J Pain 2019;23:341–53. 30144211 10.1002/ejp.1308

[13] Demoulin C Huijnen IPJ Somville P-R Grosdent S Salamun I Crielaard J-M Vanderthommen M Volders S. Relationship between different measures of pain-related fear and physical capacity of the spine in patients with chronic low back pain. Spine J 2013;13:1039–47. 23623193 10.1016/j.spinee.2013.02.037

[14] Ekman P. An argument for basic emotions. Cogn Emot 1992;6:169–200.

[15] Fairbank JC Pynsent PB. The Oswestry disability index. Spine 2000;25:2940–53. 11074683 10.1097/00007632-200011150-00017

[16] Fujii R Imai R Shigetoh H Tanaka S Morioka S. Task-specific fear influences abnormal trunk motor coordination in workers with chronic low back pain: a relative phase angle analysis of object-lifting. BMC Musculoskelet Disord 2022;23:161. 35180874 10.1186/s12891-022-05118-x PMC8857807

[17] George SZ Teyhen DS Wu SS Wright AC Dugan JL Yang G Robinson ME Childs JD. Psychosocial education improves low back pain beliefs: results from a cluster randomized clinical trial (NCT00373009) in a primary prevention setting. Eur Spine J 2009;18:1050–8. 19418075 10.1007/s00586-009-1016-7 PMC2899593

[18] Gregg CD McIntosh G Hall H Watson H Williams D Hoffman CW. The relationship between the Tampa Scale of Kinesiophobia and low back pain rehabilitation outcomes. Spine J 2015;15:2466–71. 26282104 10.1016/j.spinee.2015.08.018

[19] Gross JJ Levenson RW. Emotional suppression: physiology, self-report, and expressive behavior. J Personal Soc Psychol 1993;64:970–86. 10.1037//0022-3514.64.6.970 8326473

[20] Hapidou EG O'Brien MA Pierrynowski MR De Las Heras E Patel M Patla T. Fear and avoidance of movement in people with chronic pain: psychometric properties of the 11-item Tampa Scale for Kinesiophobia (TSK-11). Physiother Can 2012;64:235–41. 23729957 10.3138/ptc.2011-10 PMC3396571

[21] Henschke N Maher CG Refshauge KM Herbert RD Cumming RG Bleasel J York J Das A McAuley JH. Prognosis in patients with recent onset low back pain in Australian primary care: inception cohort study. BMJ 2008;337:a171. 18614473 10.1136/bmj.a171 PMC2483884

[22] Horvat M Kukolja D Ivanec D. Comparing affective responses to standardized pictures and videos: a study report. arXiv 2017. doi: 10.48550/arXiv.1505.07398.

[23] Houben RM Leeuw M Vlaeyen JW Goubert L Picavet HS. Fear of movement/injury in the general population: factor structure and psychometric properties of an adapted version of the Tampa Scale for Kinesiophobia. J Behav Med 2005;28:415–24. 16187010 10.1007/s10865-005-9011-x

[24] Knechtle D Schmid S Suter M Riner F Moschini G Senteler M Schweinhardt P Meier ML. Fear-avoidance beliefs are associated with reduced lumbar spine flexion during object lifting in pain-free adults. PAIN 2021;162:1621–31. 33323888 10.1097/j.pain.0000000000002170 PMC8120682

[25] Kori S Miller R Todd D. Kinesiophobia: a new view of chronic pain behavior. Pain Manag 1990;3:35–43.

[26] Krug RC Silva MF Lipp OV O'Sullivan PB Almeida R Peroni IS Caneiro J. An investigation of implicit bias about bending and lifting. Scand J Pain 2022;22:336–47. 34821139 10.1515/sjpain-2021-0145

[27] Leeuw M Goossens MEJB Van Breukelen GJP Boersma K Vlaeyen JWS. Measuring perceived harmfulness of physical activities in patients with chronic low back pain: the photograph series of daily activities—short electronic version. J Pain 2007;8:840–9. 17632038 10.1016/j.jpain.2007.05.013

[28] Liechti M Von Arx M Eichelberger P Bangerter C Meier ML Schmid S. Spatial distribution of erector spinae activity is related to task-specific pain-related fear during a repetitive object lifting task. J Electromyogr Kinesiol 2022;65:102678. 35696973 10.1016/j.jelekin.2022.102678

[29] Louw A Farrell K Choffin B Foster B Lunde G Snodgrass M Sweet R Weitzel M Wilder R Puentedura EJ. Immediate effect of pain neuroscience education for recent onset low back pain: an exploratory single arm trial. J Man Manipulative Ther 2019;27:267–76. 10.1080/10669817.2019.1624006 PMC6830205 31161919

[30] Matheve T De Baets L Bogaerts K Timmermans A. Lumbar range of motion in chronic low back pain is predicted by task‐specific, but not by general measures of pain‐related fear. Eur J Pain 2019;23:1171–1184. 30793429 10.1002/ejp.1384

[31] McGill SM. The biomechanics of low back injury: implications on current practice in industry and the clinic. J Biomech 1997;30:465–75. 9109558 10.1016/s0021-9290(96)00172-8

[32] Meier ML Stämpfli P Vrana A Humphreys BK Seifritz E Hotz-Boendermaker S. Neural correlates of fear of movement in patients with chronic low back pain vs. pain-free individuals. Front Hum Neurosci 2016;10:386. 27507941 10.3389/fnhum.2016.00386 PMC4960248

[33] Meier ML Vrana A Humphreys BK Seifritz E Stämpfli P Schweinhardt P. Pain-related fear—dissociable neural sources of different fear constructs. eneuro 2019;5:ENEURO.0107-0118. 10.1523/ENEURO.0107-18.2018 PMC6325558 30627654

[34] Nolan D O'Sullivan K Newton C Singh G Smith BE. Are there differences in lifting technique between those with and without low back pain? A systematic review. Scand J Pain 2020;20:215–27. 31730537 10.1515/sjpain-2019-0089

[35] Oliveira CB Franco MR Demarchi SJ Smeets RJE Huijnen IP Morelhão PK Hisamatsu TM Pinto RZ. Psychometric properties of the photograph series of daily activities-short electronic version (PHODA-SeV) in patients with chronic low back pain. J Orthop Sports Phys Ther 2018;48:719–27. 29792106 10.2519/jospt.2018.7864

[36] Oliveira CB Pinto RZ. Clinimetrics: photograph series of daily activities—short electronic version (PHODA-SeV). J Physiother 2021;67:222. 33097439 10.1016/j.jphys.2020.09.006

[37] Olsson A Nearing KI Phelps EA. Learning fears by observing others: the neural systems of social fear transmission. Soc Cogn Affect Neurosci 2007;2:3–11. 18985115 10.1093/scan/nsm005 PMC2555428

[38] Pérez-Fernández M Lerma-Lara S Ferrer-Peña R Gil-Martínez A López-de-Uralde-Villanueva I Paris-Alemany A Beltrán-Alacreu H La Touche R. Fear and difficulty perceived when visualizing therapeutic exercise in patients with chronic low back pain: a cross-sectional study. J Exerc Rehabil 2015;11:345–55. 26730386 10.12965/jer.150232 PMC4697784

[39] Saltychev M Mattie R McCormick Z Bärlund E Laimi K. Psychometric properties of the Oswestry disability index. Int J Rehabil Res 2017;40:202–8. 28368870 10.1097/MRR.0000000000000226

[40] Schaefer HS Larson CL Davidson RJ Coan JA. Brain, body, and cognition: neural, physiological and self-report correlates of phobic and normative fear. Biol Psychol 2014;98:59–69. 24561099 10.1016/j.biopsycho.2013.12.011 PMC4251669

[41] Sparto PJ Parnianpour M Reinsel TE Simon S. The effect of fatigue on multijoint kinematics and load sharing during a repetitive lifting test. Spine 1997;22:2647–54. 9399451 10.1097/00007632-199711150-00013

[42] Thomas JS France CR Lavender SA Johnson MR. Effects of fear of movement on spine velocity and acceleration after recovery from low back pain. Spine (Phila Pa 1976) 2008;33:564–70. 18317203 10.1097/BRS.0b013e3181657f1a

[43] Tkachuk GA Harris CA. Psychometric properties of the Tampa scale for kinesiophobia-11 (TSK-11). J Pain 2012;13:970–7. 23031396 10.1016/j.jpain.2012.07.001

[44] Torkar G. Pre-service teachers' fear of snakes, conservation attitudes, and likelihood of incorporating animals into the future science curriculum. J Baltic Sci Educ 2015;14:401–10.

[45] Trost Z France CR Thomas JS. Examination of the photograph series of daily activities (PHODA) scale in chronic low back pain patients with high and low kinesiophobia. PAIN 2009;141:276–82. 19131166 10.1016/j.pain.2008.11.016

[46] Trost Z France CR Vervoort T Lange JM Goubert L. Learning about pain through observation: the role of pain-related fear. J Behav Med 2014;37:257–65. 23264004 10.1007/s10865-012-9483-4

[47] Turk DC Robinson JP Sherman JJ Burwinkle T Swanson K. Assessing fear in patients with cervical pain: development and validation of the pictorial fear of activity scale-cervical (PFActS-C). PAIN 2008;139:55–62. 18417291 10.1016/j.pain.2008.03.001 PMC2580774

[48] Vianin M. Psychometric properties and clinical usefulness of the Oswestry disability index. J Chiropractic Med 2008;7:161–3. 10.1016/j.jcm.2008.07.001 PMC2697602 19646379

[49] Vlaeyen JWS Linton SJ. Fear-avoidance and its consequences in chronic musculoskeletal pain: a state of the art. PAIN 2000;85:317–32. 10781906 10.1016/S0304-3959(99)00242-0

[50] Wertli MM Rasmussen-Barr E Weiser S Bachmann LM Brunner F. The role of fear avoidance beliefs as a prognostic factor for outcome in patients with nonspecific low back pain: a systematic review. Spine J 2014;14:816–36.e4. 24412032 10.1016/j.spinee.2013.09.036

[51] Woby SR Roach NK Urmston M Watson PJ. Psychometric properties of the TSK-11: a shortened version of the Tampa Scale for Kinesiophobia. PAIN 2005;117:137–44. 16055269 10.1016/j.pain.2005.05.029

