# Supplementary material for: Tampa Scale of Kinesiophobia may underestimate task-specific fear of movement in people with and without low back pain
Source: Pain Rep. 2023 Jun 7;8(4):e1081. doi: 10.1097/PR9.0000000000001081 (PMC10247215; doi:10.1097/PR9.0000000000001081)
Supplement: SUPPLEMENTARY MATERIAL [file painreports-8-e1081-s001.pdf]

Parameter estimates from regression analysis

Hypothesis one

Table SM1: Parameter estimates of the linear mixed model between the outcome of fear and independent variables of group and methods

Parameter	Estimate	SE	df	t	P value	2.5%CI	97.5%CI
Intercept	42.448	7.266	56.376	5.842	<.001	27.896	57.001
Group = control	-2.355	9.021	56.376	-0.261	0.795	-20.424	15.714
Group = LBP	6.170	10.090	56.376	0.612	0.543	-14.040	26.380
Methods = image	3.527	4.886	95.156	0.722	0.472	-6.172	13.226
Methods = TSK-11	-8.183	8.043	54.608	-1.017	0.313	-24.303	7.938
Methods (image): Group (control)	-0.074	6.066	95.156	-0.012	0.990	-12.116	11.969
Methods (TSK-11): Group (control)	-8.173	9.986	54.608	-0.818	0.417	-28.189	11.842
Methods (image): Group (LBP)	-2.829	6.785	95.156	-0.417	0.678	-16.298	10.640
Methods (TSK-11): Group (LBP)	5.884	11.169	54.608	0.527	0.600	-16.503	28.271

Hypothesis two

Table SM2: Parameter estimates of the linear regression model between the outcome of ODI and independent variable of TSK-11 score.

Parameter	Estimate	SE	t	P value	2.5%CI	97.5%CI
Intercept	-12.209	5.556	-2.198	0.038	-23.676	-0.743
Image	0.599	0.127	4.705	<.001	0.336	0.861
Group = LBP	11.903	5.02	2.371	0.026	1.541	22.265

Table SM3: Parameter estimates of the linear regression model between the outcome of ODI and independent variable of image-based fear score.

Parameter	Estimate	SE	t	P value	2.5%CI	97.5%CI
Intercept	-12.209	5.556	-2.198	0.038	-23.676	-0.743
Image	0.599	0.127	4.705	<.001	0.336	0.861
Group = LBP	11.903	5.02	2.371	0.026	1.541	22.265

Table SM4: Parameter estimates of the linear regression model between the outcome of ODI and independent variable of video-based fear score.

Parameter	Estimate	SE	t	P value	2.5%CI	97.5%CI
Intercept	-12.209	5.556	-2.198	0.038	-23.676	-0.743
Image	0.599	0.127	4.705	<.001	0.336	0.861
Group = LBP	11.903	5.02	2.371	0.026	1.541	22.265

Hypothesis three

Table SM5: Parameter estimates of the linear mixed model between the outcome of fear and independent variables of group, methods, and load.

Parameter	Estimate	SE	df	t	P value	2.5%CI	97.5%CI
Intercept	38.935	7.777	51.537	5.006	<.001	23.326	54.544
Group = control	-3.846	9.402	48.588	-0.409	0.684	-22.745	15.053
Group = LBP	3.629	10.844	48.385	0.335	0.739	-18.171	25.428
Methods = image	-0.717	2.547	40.311	-0.282	0.78	-5.863	4.429
Load = heavy	10.961	3.161	65.689	3.467	<.001	4.648	17.273
Methods (image): Load (heavy)	4.547	4.319	137.921	1.053	0.294	-3.992	13.086
